# Bis[*O*-isopropyl (4-eth­oxy­phen­yl)dithio­phospho­nato-κ^2^ *S*,*S*′]lead(II)

**DOI:** 10.1107/S1600536812046818

**Published:** 2012-11-17

**Authors:** Shirveen Sewpersad, Werner E. Van Zyl

**Affiliations:** a School of Chemistry and Physics, University of KwaZulu-Natal, Westville Campus, Private Bag X54001, Durban 4000, South Africa

## Abstract

The title compound, [Pb(C_11_H_16_O_2_PS_2_)_2_], is a neutral four-coordinate mononuclear complex with a distorted square-pyramidal geometry of the PbS_4_ core. The apical Pb^II^ atom of each pyramid is 1.33059 (3) Å above the basal S_4_ plane. The metal atom is surrounded by two chelating dithio­phospho­nate ligands binding through the S-donor atoms. The ligands are anisobidentate as the pyramid is comprised of Pb—S bond lengths that vary substanti­ally [2.6999 (7), 2.7128 (6), 2.8877 (7) and 2.9472 (7) Å], clearly indicating two short and two longer bond lengths. The P—S bond lengths are also paired as shorter [1.9959 (9) and 1.9877 (8) Å] and slightly longer [2.0115 (9) and 2.0245 (9) Å], indicating an anisobidentate nature of the ligand whereby the shorter P—S bond has more double-bond character than the other. The S—Pb—S (chelating) bond angles range from 71.841 (18) to 72.692 (19)°, whilst the Pb—S—P bond angles range from 84.70 (3) to 90.51 (3)°.

## Related literature

For information on dithio­phospho­nate compounds, see: Van Zyl & Fackler (2000 ▶); Van Zyl (2010 ▶). For similar lead(II) dithio­phospho­nate complexes, see: Gray *et al.* (2003 ▶, 2004 ▶).
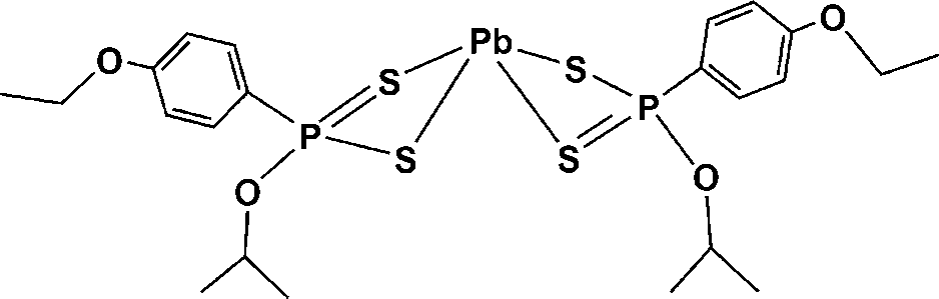


## Experimental

### 

#### Crystal data

[Pb(C_11_H_16_O_2_PS_2_)_2_]
*M* *_r_* = 757.85
Triclinic, 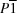

*a* = 10.2092 (8) Å
*b* = 11.7016 (10) Å
*c* = 12.9915 (10) Å
α = 89.297 (2)°
β = 85.016 (2)°
γ = 69.034 (1)°
*V* = 1443.5 (2) Å^3^
*Z* = 2
Mo *K*α radiation
μ = 6.27 mm^−1^
*T* = 173 K
0.16 × 0.14 × 0.11 mm

#### Data collection

Bruker Kappa DUO APEXII diffractometer
Absorption correction: multi-scan (*SADABS*; Sheldrick, 1996 ▶) *T*_min_ = 0.434, *T*_max_ = 0.546
36710 measured reflections
7212 independent reflections
6564 reflections with *I* > 2σ(*I*)
*R*_int_ = 0.037

#### Refinement

*R*[*F* ^2^ > 2σ(*F* ^2^)] = 0.020
*wR*(*F* ^2^) = 0.045
*S* = 1.03
7212 reflections
304 parameters
H-atom parameters constrained
Δρ_max_ = 0.61 e Å^−3^
Δρ_min_ = −0.63 e Å^−3^

### 

Data collection: *APEX2* (Bruker, 2006 ▶); cell refinement: *SAINT* (Bruker, 2006 ▶); data reduction: *SAINT*; program(s) used to solve structure: *SHELXS97* (Sheldrick, 2008 ▶); program(s) used to refine structure: *SHELXL97* (Sheldrick, 2008 ▶); molecular graphics: *SHELXTL* (Sheldrick, 2008 ▶); software used to prepare material for publication: *SHELXL97*.

## Supplementary Material

Crystal structure: contains datablock(s) I, global. DOI: 10.1107/S1600536812046818/gk2529sup1.cif

Structure factors: contains datablock(s) I. DOI: 10.1107/S1600536812046818/gk2529Isup2.hkl

Additional supplementary materials:  crystallographic information; 3D view; checkCIF report

## Figures and Tables

**Fig. 1.**
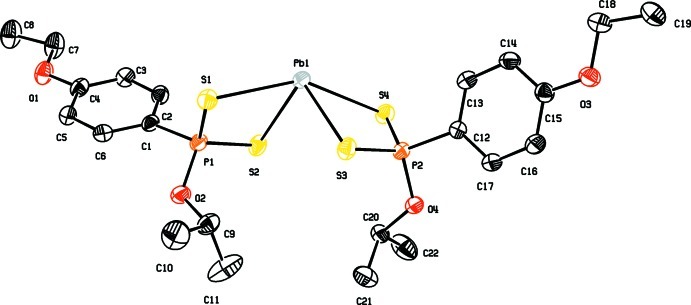
The molecular structure of the title complex with displacement ellipsoids shown at the 50% probability level. H-atoms have been omitted for clarity.

**Fig. 2.**
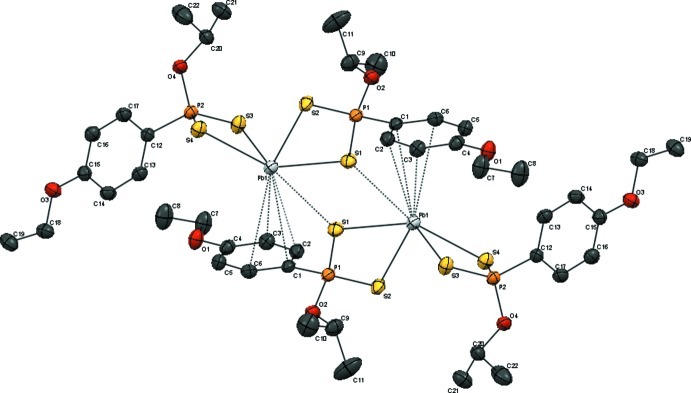
The molecular structure of a dimeric pair of the title complex showing intermolecular Pb&bull;&bull;&bull;S and Pb&bull;&bull;&bull;Ar interactions.

## supplementary crystallographic information

### Comment

The phosphor-1,1,-dithiolate class of compounds is the heavier and softer congener of the more popular phosphonate derivatives. Several sub-categories are known which include the dithiophosphato [S_2_P(OR)_2_]¯, (*R* = typically alkyl), dithiophosphinato [S_2_P*R*_2_]¯ (*R* = alkyl or aryl), and dithiophosphonato [S_2_PR(OR')]¯, (*R* = typically aryl or ferrocenyl, *R*' = alkyl) monoanionic ligands. The latter may be described as a hybrid of the former two, and are also much less developed. Lead dithiophosphonate complexes have only been developed during the past 10 years (Gray *et al.*, 2003; 2004) and the solid-state structure of the lead complexes deviate substantially from typical 4-coordinate transition-metals such as Ni(II), Cd(II), Hg(II) of the same ligand.

The title complex exhibits a structure that is built up of distorted square pyramids in dimeric pairs. The dimeric pair is characterised by intermolecular interactions between the Pb atom of one monomer with a S atom of adjacent unit, containing a Pb···S distance of 3.619 Å. Similar intermolecular interactions occur between the Pb atom of one monomeric unit and four carbon atoms in an adjacent aromatic ring in the Pb-Ar (Ar = aromatic ring) bond distance range of 3.421-3.597 Å. A related structure with a different alkoxy group was previously reported (Gray *et al.*, 2004), showing similar inter Pb···S and Pb···Ar interactions, and intermolecular Pb-S bond lenghts. It was postulated that the aforementioned Pb···Ar interactions stabilized the dimeric structure. The structure reported by Gray *et al.* (2004) adopts a similar molecular unit, the lead also resides in the centre of a distorted pyramid, 1.30 Å above the basal S_4_ plane. General and convenient methods to prepare dithiophosphonate salt derivatives have been reported (Van Zyl & Fackler, 2000).

### Experimental

A colorless methanol (40 ml) solution of NH_4_[S_2_P(O^i^Pr)(4-C_6_H_4_OEt)] (1.010 g, 3.443 mmol) was prepared. Another solution of Pb(NO_3_)_2_ (0.570 g, 1.721 mmol) in deionized water (20 ml) was prepared, and added to the colorless solution with stirring over a period of 5 min. This resulted in a white precipitate, which proved to be the formation of the title complex. The precipitate was collected by vacuum filtration, washed with water (3 *x* 10 ml) and allowed to dry under vacuum for a period of 3 hrs, yielding a dry, free-flowing white powder. Colorless crystals suitable for X-ray analysis were grown by the slow diffusion of hexane into a dichloromethane solution of the title complex. Yield: 0.926 g, 71%. M.p. 397–398 K.

^31^P NMR (CDCl_3_): δ (p.p.m.): 94.20. ^1^H NMR (CDCl_3_): δ (p.p.m.): 7.89(2*H*, dd, *o*-ArH), 6.89(2*H*, dd, *m*-ArH), 5.01(1*H*, d quart, CH), 4.03(2*H*, quart, ArOCH_2_), 1.39(3*H*, t, ArOCH_2_CH_3_), 1.32(6*H*, d, CH_3_). ^13^C NMR (CDCl_3_): δ (p.p.m.): 161.97 (*p*-ArC), 133.32 (*ipso*-Ar—C), 131.57 (*m*-ArC), 114.32 (*o*-ArC), 71.34 (CH), 64.03(ArOCH_2_), 24.85 (CH_3_), 14.91 (ArOCH_2_CH_3_).

### Refinement

All non-hydrogen atoms were refined anisotropically. All hydrogen atoms were placed in idealized positions and refined with geometrical constraints and constrained to ride on their parent atoms, with C–H = 0.95–1 00 Å,and *U*_iso_(H_aryl_) = 1.2×*U*_eq_C_aryl_ and *U*_iso_(H_methyl_) = 1.5×*U*_eq_C_methyl_.

### Crystal data

**Table:**

[Pb(C_11_H_16_O_2_PS_2_)_2_]	*Z* = 2
*M**_r_* = 757.85	*F*(000) = 744
Triclinic, *P*1	?e
Hall symbol: -P 1	*D*_x_ = 1.744 Mg m^−^^3^
*a* = 10.2092 (8) Å	Mo *K*α radiation, λ = 0.71073 Å
*b* = 11.7016 (10) Å	Cell parameters from 36710 reflections
*c* = 12.9915 (10) Å	θ = 1.6–28.4°
α = 89.297 (2)°	µ = 6.27 mm^−^^1^
β = 85.016 (2)°	*T* = 173 K
γ = 69.034 (1)°	Block, colourless
*V* = 1443.5 (2) Å^3^	0.16 × 0.14 × 0.11 mm

### Data collection

**Table:**

Bruker Kappa DUO APEXII diffractometer	7212 independent reflections
Radiation source: fine-focus sealed tube	6564 reflections with *I* > 2σ(*I*)
Graphite monochromator	*R*_int_ = 0.037
0.5° φ scans and ω scans	θ_max_ = 28.4°, θ_min_ = 1.6°
Absorption correction: multi-scan (*SADABS*; Sheldrick, 1996)	*h* = −13→13
*T*_min_ = 0.434, *T*_max_ = 0.546	*k* = −15→15
36710 measured reflections	*l* = −17→17

### Refinement

**Table:**

Refinement on *F*^2^	Secondary atom site location: difference Fourier map
Least-squares matrix: full	Hydrogen site location: inferred from neighbouring sites
*R*[*F*^2^ > 2σ(*F*^2^)] = 0.020	H-atom parameters constrained
*wR*(*F*^2^) = 0.045	*w* = 1/[σ^2^(*F*_o_^2^) + (0.0234*P*)^2^ + 0.042*P*] where *P* = (*F*_o_^2^ + 2*F*_c_^2^)/3
*S* = 1.03	(Δ/σ)_max_ = 0.002
7212 reflections	Δρ_max_ = 0.61 e Å^−^^3^
304 parameters	Δρ_min_ = −0.63 e Å^−^^3^
Primary atom site location: structure-invariant direct methods	

### Special details

**Table:**

Geometry. All e.s.d.'s (except the e.s.d. in the dihedral angle between two l.s. planes) are estimated using the full covariance matrix. The cell e.s.d.'s are taken into account individually in the estimation of e.s.d.'s in distances, angles and torsion angles; correlations between e.s.d.'s in cell parameters are only used when they are defined by crystal symmetry. An approximate (isotropic) treatment of cell e.s.d.'s is used for estimating e.s.d.'s involving l.s. planes.
Refinement. Refinement of *F*^2^ against ALL reflections. The weighted *R*-factor *wR* and goodness of fit *S* are based on *F*^2^, conventional *R*-factors *R* are based on *F*, with *F* set to zero for negative *F*^2^. The threshold expression of *F*^2^ > σ(*F*^2^) is used only for calculating *R*-factors(gt) *etc*. and is not relevant to the choice of reflections for refinement. *R*-factors based on *F*^2^ are statistically about twice as large as those based on *F*, and *R*- factors based on ALL data will be even larger.

### Fractional atomic coordinates and isotropic or equivalent isotropic displacement parameters (Å^2^)

**Table:**

	*x*	*y*	*z*	*U*_iso_*/*U*_eq_	
Pb1	0.563161 (9)	0.892734 (7)	0.676272 (7)	0.02692 (3)	
S1	0.48642 (6)	0.83844 (6)	0.47888 (5)	0.03000 (13)	
S2	0.80447 (7)	0.77787 (6)	0.55716 (5)	0.03150 (13)	
S3	0.55200 (7)	0.68031 (6)	0.75505 (5)	0.03173 (14)	
S4	0.75140 (7)	0.82120 (5)	0.84420 (5)	0.03020 (13)	
P1	0.69313 (6)	0.77422 (5)	0.43769 (5)	0.02499 (13)	
P2	0.67269 (7)	0.68943 (5)	0.86809 (5)	0.02494 (13)	
O1	0.8185 (2)	1.06377 (17)	0.08990 (14)	0.0368 (4)	
O2	0.74190 (18)	0.64074 (14)	0.38776 (13)	0.0314 (4)	
O3	0.31553 (18)	0.75446 (15)	1.26420 (13)	0.0315 (4)	
O4	0.79180 (18)	0.55982 (14)	0.88024 (13)	0.0299 (4)	
C1	0.7390 (2)	0.8566 (2)	0.33187 (18)	0.0244 (5)	
C2	0.7854 (2)	0.9519 (2)	0.35221 (19)	0.0280 (5)	
H2	0.7992	0.9676	0.4213	0.034*	
C3	0.8118 (3)	1.0243 (2)	0.27399 (19)	0.0295 (5)	
H3	0.8416	1.0898	0.2894	0.035*	
C4	0.7941 (3)	1.0000 (2)	0.17254 (19)	0.0293 (5)	
C5	0.7474 (3)	0.9048 (2)	0.15114 (19)	0.0294 (5)	
H5	0.7350	0.8885	0.0819	0.035*	
C6	0.7191 (3)	0.8344 (2)	0.22993 (18)	0.0276 (5)	
H6	0.6861	0.7707	0.2148	0.033*	
C7	0.8657 (3)	1.1634 (3)	0.1081 (2)	0.0438 (7)	
H7A	0.9567	1.1329	0.1394	0.053*	
H7B	0.7958	1.2251	0.1558	0.053*	
C8	0.8824 (4)	1.2188 (3)	0.0058 (2)	0.0507 (8)	
H8A	0.9426	1.1547	−0.0432	0.076*	
H8B	0.9257	1.2803	0.0139	0.076*	
H8C	0.7898	1.2578	−0.0203	0.076*	
C9	0.7074 (3)	0.5436 (2)	0.4444 (2)	0.0361 (6)	
H9	0.6554	0.5776	0.5125	0.043*	
C10	0.6158 (4)	0.5027 (3)	0.3833 (3)	0.0567 (9)	
H10A	0.5333	0.5735	0.3681	0.085*	
H10B	0.5850	0.4437	0.4230	0.085*	
H10C	0.6684	0.4639	0.3185	0.085*	
C11	0.8454 (4)	0.4454 (3)	0.4627 (3)	0.0702 (11)	
H11A	0.8952	0.4093	0.3962	0.105*	
H11B	0.8281	0.3817	0.5054	0.105*	
H11C	0.9029	0.4810	0.4983	0.105*	
C12	0.5720 (3)	0.7089 (2)	0.99137 (18)	0.0251 (5)	
C13	0.4855 (3)	0.8252 (2)	1.02436 (19)	0.0294 (5)	
H13	0.4853	0.8933	0.9837	0.035*	
C14	0.3992 (3)	0.8444 (2)	1.11533 (19)	0.0297 (5)	
H14	0.3414	0.9251	1.1376	0.036*	
C15	0.3976 (3)	0.7450 (2)	1.17388 (18)	0.0269 (5)	
C16	0.4827 (3)	0.6269 (2)	1.14110 (19)	0.0307 (5)	
H16	0.4811	0.5588	1.1811	0.037*	
C17	0.5694 (3)	0.6088 (2)	1.05057 (19)	0.0297 (5)	
H17	0.6275	0.5281	1.0283	0.036*	
C18	0.2206 (3)	0.8742 (2)	1.2982 (2)	0.0331 (6)	
H18A	0.1523	0.9098	1.2464	0.040*	
H18B	0.2738	0.9290	1.3069	0.040*	
C19	0.1447 (3)	0.8615 (2)	1.3993 (2)	0.0375 (6)	
H19A	0.0849	0.8141	1.3885	0.056*	
H19B	0.0861	0.9429	1.4273	0.056*	
H19C	0.2134	0.8193	1.4481	0.056*	
C20	0.9080 (3)	0.5123 (2)	0.79867 (19)	0.0305 (5)	
H20	0.8964	0.5734	0.7425	0.037*	
C21	0.9019 (3)	0.3954 (2)	0.7559 (2)	0.0418 (7)	
H21A	0.9166	0.3347	0.8105	0.063*	
H21B	0.9756	0.3638	0.6990	0.063*	
H21C	0.8095	0.4117	0.7304	0.063*	
C22	1.0424 (3)	0.4935 (3)	0.8471 (3)	0.0529 (8)	
H22A	1.0400	0.5717	0.8750	0.079*	
H22B	1.1225	0.4615	0.7948	0.079*	
H22C	1.0525	0.4349	0.9032	0.079*	

### Atomic displacement parameters (Å^2^)

**Table:**

	*U*^11^	*U*^22^	*U*^33^	*U*^12^	*U*^13^	*U*^23^
Pb1	0.02779 (5)	0.02345 (5)	0.02595 (5)	−0.00485 (3)	−0.00277 (4)	0.00404 (3)
S1	0.0212 (3)	0.0358 (3)	0.0304 (3)	−0.0072 (2)	−0.0021 (2)	0.0031 (2)
S2	0.0231 (3)	0.0399 (3)	0.0279 (3)	−0.0064 (2)	−0.0058 (2)	0.0074 (3)
S3	0.0417 (4)	0.0317 (3)	0.0279 (3)	−0.0191 (3)	−0.0099 (3)	0.0051 (2)
S4	0.0366 (4)	0.0277 (3)	0.0305 (3)	−0.0156 (3)	−0.0072 (3)	0.0037 (2)
P1	0.0224 (3)	0.0250 (3)	0.0244 (3)	−0.0050 (2)	−0.0013 (2)	0.0044 (2)
P2	0.0297 (3)	0.0213 (3)	0.0233 (3)	−0.0084 (2)	−0.0036 (2)	0.0030 (2)
O1	0.0457 (11)	0.0417 (10)	0.0306 (10)	−0.0245 (9)	−0.0069 (8)	0.0114 (8)
O2	0.0349 (10)	0.0250 (8)	0.0300 (9)	−0.0067 (7)	0.0021 (8)	0.0039 (7)
O3	0.0326 (10)	0.0268 (8)	0.0324 (10)	−0.0093 (7)	0.0048 (8)	−0.0016 (7)
O4	0.0327 (10)	0.0239 (8)	0.0265 (9)	−0.0035 (7)	0.0023 (7)	0.0038 (7)
C1	0.0196 (11)	0.0246 (11)	0.0244 (12)	−0.0028 (8)	−0.0013 (9)	0.0046 (9)
C2	0.0258 (13)	0.0320 (12)	0.0251 (12)	−0.0086 (10)	−0.0040 (10)	0.0018 (9)
C3	0.0290 (13)	0.0303 (12)	0.0322 (13)	−0.0139 (10)	−0.0052 (11)	0.0027 (10)
C4	0.0263 (13)	0.0306 (12)	0.0297 (13)	−0.0090 (10)	−0.0020 (10)	0.0088 (10)
C5	0.0316 (14)	0.0305 (12)	0.0249 (12)	−0.0093 (10)	−0.0043 (10)	0.0027 (10)
C6	0.0264 (13)	0.0244 (11)	0.0306 (13)	−0.0075 (9)	−0.0024 (10)	0.0011 (9)
C7	0.0547 (19)	0.0535 (17)	0.0378 (16)	−0.0360 (15)	−0.0109 (14)	0.0127 (13)
C8	0.070 (2)	0.0589 (19)	0.0415 (17)	−0.0446 (17)	−0.0103 (16)	0.0164 (14)
C9	0.0384 (15)	0.0304 (13)	0.0401 (15)	−0.0135 (11)	−0.0029 (12)	0.0108 (11)
C10	0.062 (2)	0.057 (2)	0.060 (2)	−0.0307 (17)	−0.0116 (18)	0.0045 (16)
C11	0.062 (2)	0.0476 (19)	0.106 (3)	−0.0207 (17)	−0.034 (2)	0.035 (2)
C12	0.0282 (13)	0.0230 (10)	0.0224 (11)	−0.0070 (9)	−0.0020 (10)	0.0008 (9)
C13	0.0328 (14)	0.0227 (11)	0.0307 (13)	−0.0074 (10)	−0.0035 (11)	0.0042 (9)
C14	0.0311 (13)	0.0215 (11)	0.0333 (13)	−0.0061 (9)	−0.0006 (11)	−0.0008 (9)
C15	0.0281 (13)	0.0278 (11)	0.0254 (12)	−0.0104 (9)	−0.0037 (10)	−0.0013 (9)
C16	0.0361 (14)	0.0250 (11)	0.0309 (13)	−0.0116 (10)	−0.0007 (11)	0.0043 (10)
C17	0.0336 (14)	0.0220 (11)	0.0298 (13)	−0.0058 (9)	−0.0009 (11)	0.0017 (9)
C18	0.0295 (14)	0.0289 (12)	0.0380 (15)	−0.0071 (10)	−0.0007 (11)	−0.0054 (10)
C19	0.0309 (14)	0.0423 (14)	0.0382 (15)	−0.0132 (11)	0.0042 (12)	−0.0093 (12)
C20	0.0346 (14)	0.0275 (12)	0.0257 (12)	−0.0080 (10)	0.0037 (11)	−0.0012 (9)
C21	0.0446 (17)	0.0352 (14)	0.0438 (16)	−0.0140 (12)	0.0062 (13)	−0.0100 (12)
C22	0.0365 (17)	0.065 (2)	0.0524 (19)	−0.0132 (15)	0.0009 (15)	−0.0208 (16)

### Geometric parameters (Å, º)

**Table:**

Pb1—S2	2.6999 (7)	C9—C10	1.480 (4)
Pb1—S3	2.7128 (6)	C9—C11	1.502 (4)
Pb1—S1	2.8877 (7)	C9—H9	1.0000
Pb1—S4	2.9472 (7)	C10—H10A	0.9800
S1—P1	1.9959 (9)	C10—H10B	0.9800
S2—P1	2.0115 (9)	C10—H10C	0.9800
S3—P2	2.0245 (9)	C11—H11A	0.9800
S4—P2	1.9877 (8)	C11—H11B	0.9800
P1—O2	1.5877 (17)	C11—H11C	0.9800
P1—C1	1.794 (2)	C12—C13	1.380 (3)
P2—O4	1.5848 (16)	C12—C17	1.400 (3)
P2—C12	1.797 (2)	C13—C14	1.381 (3)
O1—C4	1.356 (3)	C13—H13	0.9500
O1—C7	1.441 (3)	C14—C15	1.386 (3)
O2—C9	1.476 (3)	C14—H14	0.9500
O3—C15	1.363 (3)	C15—C16	1.392 (3)
O3—C18	1.437 (3)	C16—C17	1.379 (3)
O4—C20	1.471 (3)	C16—H16	0.9500
C1—C2	1.394 (3)	C17—H17	0.9500
C1—C6	1.399 (3)	C18—C19	1.500 (4)
C2—C3	1.384 (3)	C18—H18A	0.9900
C2—H2	0.9500	C18—H18B	0.9900
C3—C4	1.392 (3)	C19—H19A	0.9800
C3—H3	0.9500	C19—H19B	0.9800
C4—C5	1.398 (3)	C19—H19C	0.9800
C5—C6	1.382 (3)	C20—C22	1.503 (4)
C5—H5	0.9500	C20—C21	1.507 (3)
C6—H6	0.9500	C20—H20	1.0000
C7—C8	1.495 (4)	C21—H21A	0.9800
C7—H7A	0.9900	C21—H21B	0.9800
C7—H7B	0.9900	C21—H21C	0.9800
C8—H8A	0.9800	C22—H22A	0.9800
C8—H8B	0.9800	C22—H22B	0.9800
C8—H8C	0.9800	C22—H22C	0.9800
			
S2—Pb1—S3	93.09 (2)	C11—C9—H9	109.0
S2—Pb1—S1	72.692 (19)	C9—C10—H10A	109.5
S3—Pb1—S1	91.516 (19)	C9—C10—H10B	109.5
S2—Pb1—S4	82.708 (19)	H10A—C10—H10B	109.5
S3—Pb1—S4	71.841 (18)	C9—C10—H10C	109.5
S1—Pb1—S4	149.566 (17)	H10A—C10—H10C	109.5
P1—S1—Pb1	85.24 (3)	H10B—C10—H10C	109.5
P1—S2—Pb1	90.16 (3)	C9—C11—H11A	109.5
P2—S3—Pb1	90.51 (3)	C9—C11—H11B	109.5
P2—S4—Pb1	84.70 (3)	H11A—C11—H11B	109.5
O2—P1—C1	100.90 (10)	C9—C11—H11C	109.5
O2—P1—S1	110.94 (7)	H11A—C11—H11C	109.5
C1—P1—S1	111.88 (8)	H11B—C11—H11C	109.5
O2—P1—S2	111.34 (7)	C13—C12—C17	119.0 (2)
C1—P1—S2	109.69 (8)	C13—C12—P2	118.89 (18)
S1—P1—S2	111.63 (4)	C17—C12—P2	121.87 (17)
O4—P2—C12	101.33 (10)	C12—C13—C14	121.2 (2)
O4—P2—S4	112.17 (8)	C12—C13—H13	119.4
C12—P2—S4	111.43 (8)	C14—C13—H13	119.4
O4—P2—S3	109.88 (7)	C13—C14—C15	119.5 (2)
C12—P2—S3	109.56 (9)	C13—C14—H14	120.2
S4—P2—S3	111.98 (4)	C15—C14—H14	120.2
C4—O1—C7	118.0 (2)	O3—C15—C14	123.9 (2)
C9—O2—P1	119.79 (15)	O3—C15—C16	116.0 (2)
C15—O3—C18	117.87 (18)	C14—C15—C16	120.1 (2)
C20—O4—P2	119.72 (14)	C17—C16—C15	119.9 (2)
C2—C1—C6	118.8 (2)	C17—C16—H16	120.0
C2—C1—P1	119.26 (18)	C15—C16—H16	120.0
C6—C1—P1	121.73 (18)	C16—C17—C12	120.2 (2)
C3—C2—C1	121.4 (2)	C16—C17—H17	119.9
C3—C2—H2	119.3	C12—C17—H17	119.9
C1—C2—H2	119.3	O3—C18—C19	108.0 (2)
C2—C3—C4	119.3 (2)	O3—C18—H18A	110.1
C2—C3—H3	120.4	C19—C18—H18A	110.1
C4—C3—H3	120.4	O3—C18—H18B	110.1
O1—C4—C3	124.3 (2)	C19—C18—H18B	110.1
O1—C4—C5	115.9 (2)	H18A—C18—H18B	108.4
C3—C4—C5	119.8 (2)	C18—C19—H19A	109.5
C6—C5—C4	120.5 (2)	C18—C19—H19B	109.5
C6—C5—H5	119.8	H19A—C19—H19B	109.5
C4—C5—H5	119.8	C18—C19—H19C	109.5
C5—C6—C1	120.1 (2)	H19A—C19—H19C	109.5
C5—C6—H6	119.9	H19B—C19—H19C	109.5
C1—C6—H6	119.9	O4—C20—C22	107.2 (2)
O1—C7—C8	107.2 (2)	O4—C20—C21	107.8 (2)
O1—C7—H7A	110.3	C22—C20—C21	112.9 (2)
C8—C7—H7A	110.3	O4—C20—H20	109.6
O1—C7—H7B	110.3	C22—C20—H20	109.6
C8—C7—H7B	110.3	C21—C20—H20	109.6
H7A—C7—H7B	108.5	C20—C21—H21A	109.5
C7—C8—H8A	109.5	C20—C21—H21B	109.5
C7—C8—H8B	109.5	H21A—C21—H21B	109.5
H8A—C8—H8B	109.5	C20—C21—H21C	109.5
C7—C8—H8C	109.5	H21A—C21—H21C	109.5
H8A—C8—H8C	109.5	H21B—C21—H21C	109.5
H8B—C8—H8C	109.5	C20—C22—H22A	109.5
O2—C9—C10	108.7 (2)	C20—C22—H22B	109.5
O2—C9—C11	106.4 (2)	H22A—C22—H22B	109.5
C10—C9—C11	114.4 (3)	C20—C22—H22C	109.5
O2—C9—H9	109.0	H22A—C22—H22C	109.5
C10—C9—H9	109.0	H22B—C22—H22C	109.5
			
S2—Pb1—S1—P1	−3.39 (3)	C6—C1—C2—C3	0.0 (3)
S3—Pb1—S1—P1	−96.16 (3)	P1—C1—C2—C3	−175.01 (18)
S4—Pb1—S1—P1	−40.94 (5)	C1—C2—C3—C4	−1.2 (4)
S3—Pb1—S2—P1	93.98 (3)	C7—O1—C4—C3	−0.1 (4)
S1—Pb1—S2—P1	3.36 (3)	C7—O1—C4—C5	179.5 (2)
S4—Pb1—S2—P1	165.23 (3)	C2—C3—C4—O1	−179.1 (2)
S2—Pb1—S3—P2	87.41 (3)	C2—C3—C4—C5	1.3 (4)
S1—Pb1—S3—P2	160.16 (3)	O1—C4—C5—C6	−179.9 (2)
S4—Pb1—S3—P2	6.13 (3)	C3—C4—C5—C6	−0.2 (4)
S2—Pb1—S4—P2	−101.96 (3)	C4—C5—C6—C1	−0.9 (4)
S3—Pb1—S4—P2	−6.27 (3)	C2—C1—C6—C5	1.0 (3)
S1—Pb1—S4—P2	−66.05 (4)	P1—C1—C6—C5	175.93 (18)
Pb1—S1—P1—O2	129.49 (7)	C4—O1—C7—C8	−179.2 (2)
Pb1—S1—P1—C1	−118.68 (9)	P1—O2—C9—C10	117.6 (2)
Pb1—S1—P1—S2	4.68 (3)	P1—O2—C9—C11	−118.6 (2)
Pb1—S2—P1—O2	−129.58 (7)	O4—P2—C12—C13	160.0 (2)
Pb1—S2—P1—C1	119.60 (8)	S4—P2—C12—C13	40.5 (2)
Pb1—S2—P1—S1	−4.99 (4)	S3—P2—C12—C13	−84.0 (2)
Pb1—S4—P2—O4	132.71 (7)	O4—P2—C12—C17	−25.8 (2)
Pb1—S4—P2—C12	−114.48 (9)	S4—P2—C12—C17	−145.23 (19)
Pb1—S4—P2—S3	8.62 (4)	S3—P2—C12—C17	90.3 (2)
Pb1—S3—P2—O4	−134.69 (7)	C17—C12—C13—C14	1.4 (4)
Pb1—S3—P2—C12	114.82 (8)	P2—C12—C13—C14	175.8 (2)
Pb1—S3—P2—S4	−9.33 (4)	C12—C13—C14—C15	−1.1 (4)
C1—P1—O2—C9	−172.27 (18)	C18—O3—C15—C14	2.8 (3)
S1—P1—O2—C9	−53.57 (19)	C18—O3—C15—C16	−176.9 (2)
S2—P1—O2—C9	71.40 (18)	C13—C14—C15—O3	−179.5 (2)
C12—P2—O4—C20	−174.11 (17)	C13—C14—C15—C16	0.2 (4)
S4—P2—O4—C20	−55.17 (18)	O3—C15—C16—C17	−179.9 (2)
S3—P2—O4—C20	70.07 (17)	C14—C15—C16—C17	0.4 (4)
O2—P1—C1—C2	−145.78 (18)	C15—C16—C17—C12	0.0 (4)
S1—P1—C1—C2	96.21 (19)	C13—C12—C17—C16	−0.8 (4)
S2—P1—C1—C2	−28.2 (2)	P2—C12—C17—C16	−175.1 (2)
O2—P1—C1—C6	39.3 (2)	C15—O3—C18—C19	−178.9 (2)
S1—P1—C1—C6	−78.67 (19)	P2—O4—C20—C22	119.9 (2)
S2—P1—C1—C6	156.88 (17)	P2—O4—C20—C21	−118.3 (2)
